# Donor Complications Following Laparoscopic Compared to Hand-Assisted Living Donor Nephrectomy: An Analysis of the Literature

**DOI:** 10.1155/2010/825689

**Published:** 2010-01-06

**Authors:** Whitney R. Halgrimson, Jeffrey Campsen, M. Susan Mandell, Mara A. Kelly, Igal Kam, Michael A. Zimmerman

**Affiliations:** ^1^Division of Transplant Surgery, University of Colorado Health Sciences Center, P.O. Box 6510, Aurora, CO 80045I, USA; ^2^Department of Anesthesia, University of Colorado Health Sciences Center, P.O. Box 6510, Aurora, CO 80045I, USA

## Abstract

There are two approaches to laparoscopic donor nephrectomy: standard laparoscopic donor nephrectomy (LDN) and hand-assisted laparoscopic donor nephrectomy (HALDN). In this study we report the operative statistics and donor complications associated with LDN and HALDN from large-center peer-reviewed publications. *Methods*. We conducted PubMed and Ovid searches to identify LDN and HALDN outcome studies that were published after 2004. *Results*. There were 37 peer-reviewed studies, each with more than 150 patients. Cumulatively, over 9000 patients were included in this study. LDN donors experienced a higher rate of intraoperative complications than HALDN donors (5.2% versus. 2.0%, *P* < .001). Investigators did not report a significant difference in the rate of major postoperative complications between the two groups (LDN 0.5% versus HALDN 0.7%, *P* = .111). However, conversion to open procedures from vascular injury was reported more frequently in LDN procedures (0.8% versus 0.4%, *P* = .047). *Conclusion*. At present there is no evidence to support the use of one laparoscopic approach in preference to the other. There are trends in the data suggesting that intraoperative injuries are more common in LDN while minor postoperative complications are more common in HALDN.

## 1. Introduction

There has been a rapid increase in living organ donation in order to bridge the shortfall between the demand and supply of donor organs. Kidney transplant recipients have benefited considerably by the expansion in living donation. The 2007 annual report from the Scientific Registry of Transplant Recipients (SRTR) shows that 45% of all transplanted kidneys were obtained from living donors [1]. Until 1995, donor nephrectomy surgery was performed through a large flank incision. Subsequently, laparoscopic donor nephrectomy (LDN) became the surgical approach of choice [2]. 

Evidence shows that LDN does not adversely affect donor graft function or survival compared to kidney recovery through an open approach [3]. Laparoscopic techniques also reduce length of hospitalization, improve pain scores, and produce a better cosmetic outcome [4–7]. Nonetheless, LDN requires additional technical training with a distinct learning curve, and surgeons may initially experience longer operating and warm ischemic times [5, 8, 9]. Published reports also suggest that LDN is more technically challenging than open donor nephrectomy (ODN) in patients who are greater than their ideal body weight [2]. 

Surgeons have modified the technique of LDN in order to make the recovery of the donor kidney easier and safer. One approach uses a longer incision at the extraction site so that the surgeon's hand can be inserted into the peritoneal cavity to assist with laparoscopic donor nephrectomy [10, 11]. Some investigators report shorter warm ischemic times and less intraoperative bleeding with hand-assisted laparoscopic nephrectomy (HALDN) compared to LDN [12]. HALDN has also been used in donors who were previously considered poor candidates for LDN, including those who weigh more than their ideal body weight, who have anatomic variations in the renal vasculature or who had previous abdominal surgery [12]. Generally the rate of complications appears to be similar between HALN and LDN. However, the type and severity of complications has not been systematically compared.

Currently the choice of procedure largely depends on the personal preference and experience of the surgeon [2, 13]. A 2008 survey of transplant fellowship training programs reported that 41% of responding centers preferred LDN, 22% preferred HALDN, and 37% used a combination of LDN and HALDN procedures [2]. The variability in practice can be partly attributed to a lack of detail about the risks associated with both techniques [12]. In this study we report summarized information on the rate and type of donor complications derived from large retro- and prospective institutional publications on LDN and HALDN.

## 2. Methods

### 2.1. Literature Search

We performed Pubmed and Ovid keyword searches to identify all studies on LDN and HALDN. Search words included laparoscopic donor nephrectomy, LDN, and HALDN. We included all literature that was published between January 01, 2004 and April 30, 2009. The inclusion criteria for our review were studies that enlisted adult patients, only, described a transperitoneal surgical technique, and were published in English. Studies were included that described outcomes in greater than 150 LDN patients or 50 HALDN patients or studies that directly compared outcomes between the two groups in any number of patients. A total of 37 studies were included in the final analysis. A total of 206 studies were excluded because they did not use standard laparoscopic or hand-assisted approaches (e.g., retroperitoneal approach, robot-assistance, etc.), they were review or editorial publications, or they did not contain specific information on donor outcomes. In cases where one study population was contained in more than one publication, we analyzed the most recently published report that included the largest study cohort and that focused on donor outcomes.

### 2.2. Data Collection

We were unable to construct a standard metadatabank for analysis because the design of all the studies uncovered by the search was descriptive. Therefore studies lacked standard techniques for validity assessment and data abstraction such as blinding, reliability checks, or duplicate observations. In addition, we accepted more than one type of data collection method, including retrospective, prospective, and randomized controlled. We therefore could not use meta-analysis and chose instead to summate the data in order to provide an estimate of the risks and complications associated with LDN compared to HALDN. 

 We summated the data for the categories of LDN and for HALDN and recorded eight distinct patient and outcome variables. These included age, body mass index (BMI), total operative time, estimated blood loss, warm ischemic time, length of hospital stay, intraoperative and postoperative complications. Deaths during the admission hospitalization were also recorded.

### 2.3. Data Analysis

Discrete variables were recorded as the number of reported events, while continuous measures (age, BMI, total donor operative time, estimated blood loss, warm ischemia time, length of hospital stay) were expressed as the weighted mean of the reported arithmetic means in the dataset. Weighted mean calculations were based on sample size due to the lack of reporting of the standard error from many studies. In studies that did not explicitly define their measure of total donor operative time we used a measure from skin incision to skin closure. Twenty of the 34 studies that reported total operative time did not give a specific definition, and three studies were excluded from the calculation of operative time because they used alternative endpoints in their definition. Similarly, we estimated warm ischemia time (WIT) from renal vessel occlusion to immersion in ice or back-table perfusion if this information was not included. Seventeen of 40 studies reporting WIT did not give an explicit definition but did provide enough information to estimate the WIT using the above definition.

We identified complications that represented significant donor morbidity or mortality and led to an escalation in care. These were blood transfusion, aborted procedure, conversion to open surgical procedure, rehospitalization, reoperation, and death. Complication rates were compared using a two-tailed z test of two binomial proportions. The calculated weighted means were compared using the “glm” procedure function in SAS software.

## 3. Results

### 3.1. Database

The search produced 37 studies that met all the inclusion criteria. Nine of the 37 studies reported and compared outcomes of both HALDN and LDN. Twelve described the outcomes of only HALDN outcomes and 16 described only LDN outcomes. There were 26 retrospective, eight prospective, and one randomized control trial. Two studies did not clearly define their study design. The dataset derived from the 37 publications included 9296 patients, of which 2562 were HALDN and 6734 were LDN. Details of the study design are reported in Table 1. The intraoperative and post-operative donor complications were classified into categories based on the organ system that was injured and if the injury was related to procedural problems. The classification of the intra and post-operative complications is shown in Tables 2 and 3.

Patient demographics and intraoperative weighted mean values are displayed in Table 1, although we could not extract complete information in all the analytic categories. Of the 37 studies, 34 reported total operative time, 22 reported estimated blood loss, 30 reported WIT, and 29 reported length of stay. In addition, the BMI of donor nephrectomy patients was only reported in 18 studies, making it the least reported variable. 

The average age of LDN donors was 39.9 years while that of the HALDN donors was 41.2 years. Donors had similar average BMI (26.8 kg/m^2^ LDN versus 27.3 kg/m^2^ HALDN). Mean operative time was 187.0 minutes in LDN procedures and 189.3 minutes in HALDN procedures. Mean length of stay was slightly shorter but not significantly different for LDN donors (2.7 days versus 3.0 days). Conversely, HALDN donor warm ischemic time was shorter (3.7 minutes versus 2.5 minutes) and estimated blood loss was less (144.0 mL versus 128.6 mL). However, only WIT was found to be significantly different between LDN and HALDN cases (*P* = .037).

### 3.2. Intraoperative Complications

The rate of intraoperative complications was significantly higher in LDN donors compared to HALDN donors (5.2% versus 2.0%, *P* < .001). Vascular injury or bleeding, bowel injury, pulmonary injury, and liver, mesenteric or splenic injury were significantly more common with LDN (Table 2). There was also a greater rate of technical difficulties encountered during surgery with LDN. We did not find a significant difference in the incidence of renal or ureteral injury.

The LDN rate of vascular injury or bleeding was 2.0% while the HALDN rate was only 1.1% (*P* = .003). Within the LDN group there was also a higher rate of major bleeding (defined as greater than two units of packed red blood cells or 500 mL blood loss). The rate of bleeding due to other vascular injuries was also elevated in LDN donors, but we did not find a statistically significant difference when compared with HALDN (1.4% versus 0.9%, *P* = .056). LDN donors also experienced significantly higher rates of bowel injury (0.4% versus 0.1%, *P* = .031), and liver, mesenteric or splenic injury (0.7% versus 0.2%, *P* = .001).

Procedural complications occurred more frequently in LDN than in HALDN cases (0.8% versus 0.4%, *P* = .008). Most complications were reported as difficult manual extractions, entrapment sack malfunctions, or elective conversions to open procedures. Only difficult manual extractions and entrapment sack malfunctions were found to be significantly elevated (0.2% versus 0.0%, *P* = .033).

### 3.3. Postoperative Complications

The incidence of post-operative complications in LDN donors was statistically lower than in HALDN donors (6.7% versus 8.6%, *P* = .002) (Table 3). There was no statistical difference found between the summated rates of cardiac, neural, pulmonary, or vascular complications. However, we observed striking differences between LDN and HALDN rates of incisional, renal, and ureteric complications. Incision and wound complications occurred in 1.7% of LDN donors compared to 3.1% of HALDN donors (*P* < .001). Notably, LDN had lower rates of incisional hernias (0.1% versus 0.7%, *P* < .001) and wound infections (1.4% versus 2.0%, *P* = .034). The rates of abdominal and bowel complications were also found to be significantly different. There were no statistical differences in other complications listed in this category. 

LDN donors had a markedly lower incidence of renal and ureteric complications (0.4% versus 1.6%, *P* < .001). This finding was explained by the larger number of HALDN patients who suffered from urinary retention and urinary tract infections (both 0.2% LDN versus 0.7% HALDN, *P* < .001). The rate of post-operative ileus was significantly greater in HALDN donors than in LDN donors (0.5% versus 1.0%, *P* = .008). This elevated ileus rate was the primary determinant of overall bowel complications in HALDN groups (0.7% versus 1.1%, *P* = .025).

The LDN and HALDN donors experienced similar rates of post-operative vascular complications and hematomas, 0.9% versus 0.7%, respectively (*P* = .525). However, there were significant differences within this category. The rates of subcapsular hematoma and continued bleeding were statistically greater in the LDN donors (*P* = .026). Conversely, only HALDN donors experienced pulmonary embolism or deep vein thrombus (0.0% versus 0.2%, *P* = .001).

### 3.4. Major Complications

The summated rate of major intraoperative complications was significantly higher in LDN donors than in HALDN donors (2.2% versus 1.1%, *P* = .001). This difference was due to major injuries that required blood transfusions or conversion to an open procedure. Blood transfusions were needed in 0.8% of LDN donors versus only 0.2% of HALDN donors (*P* = .004). The overall rate of conversions to open surgery was also significantly higher in LDN patients (1.3% versus 0.8%, *P* = .030), primarily due to bleeding (0.8% versus 0.4%, *P* = .047).

We did not find a significant difference in the rate of conversions to an open procedure due to obesity or other reasons. Even though there were two aborted procedures in the LDN group, due to a colon and a mesenteric vein injury, no significant difference was detected between the two groups. 

The rate of major post-operative complications was similar in HALDN donors (0.5% versus 0.7%, *P* = .111). Significant differences were only observed in a higher incidence of rehospitalization among HALDN donors (0.3%) versus LDN donors (0.1%) (*P* = .007). Ileus was the most common reported reason for rehospitalization. Reoperations were needed in 0.4% of both types of laparoscopic procedure (*P* = .807). The indications for additional surgery included bleeding or hematoma, bowel injury, small bowel obstruction, and incisional hernias. Two patients died perioperatively: one HALDN donor died due to a thromboembolism and one LDN donor died due to a myocardial infarction.

The overall rate of major intraoperative and post-operative complications was 2.6% in LDN donors and was 1.8% in HALDN donors. The difference was found to be statistically significant (*P* = .013). The relative rates of all major complications are found in Table 4.

## 4. Discussion

Living donor nephrectomy is unique in that the donor assumes an operative risk yet has no underlying disease and does not have any direct medical benefit from the procedure. This explains the clear imperative to ensure that donor complications are minimized [3, 40]. While LDN and HALDN techniques have been shown to be safe compared to open surgery, both techniques appear to have different rates and types of complications. The data we analyzed suggest that LDN donors experience higher rates of intraoperative complications than HALDN donors. Furthermore, the incidence of major bleeding and overall vascular injury was also greater in the LDN group and more commonly required open conversion and blood transfusions. Conversely, the rate of postoperative wound infections renal and ureteric injuries appeared to be greater in the HALDN group. 

While proponents of HALDN point out the advantages of a kidney extraction site and improved tactile control during the procedure, investigators have questioned if HALDN patients suffer from a greater number of post-operative complications [50]. Our data analysis strongly supports the impression that HALDN donors have greater incision morbidity than LDN donors. This contrasts with the findings of Kocak et al., who reported no significant differences in incision morbidity in a large direct comparison of LDN and HALDN donor complications [35]. We have no data to explain this discrepancy, but it may suggest that institutional practices and technical experiences play a role in determining outcomes unique to each study. We did not find significant differences in the rate of re-hospitalization due to infection nor the rate of re-operation for incisional hernia. 

Post-operative bowel complications were cited as significant sources of donor morbidity in HALDN and thus are the reason why some centers choose to employ the LDN technique in favor of HALDN [35]. We identified greater rates of ileus in HALDN donors and re-hospitalization due to ileus. Unexpectedly, post-operative renal and ureteric complications were also significantly elevated among HALDN donors. This may be a concern and may motivate surgeons to select the LDN in preference to the HALDN technique. However, we found that 50% of observations came from one HALDN trial [25] and 25% came from another [29]. Therefore the increased rate of ureteric and renal complications could be due to unidentified center specific practices that are not found at other institutions.

There is debate in the literature if one laparoscopic technique is preferential to the other in obese donors. Heimbach et al. found that HALDN was safe in obese donors (BMI>30 kg/m^2^); however total operative times and intraoperative complications were increased in significantly obese donors [29]. In contrast, Sundaram et al. did not find significantly elevated operative or post-operative complication rates in obese LDN donors [49]. In our limited analysis, we found that obese LDN and HALDN donors had nearly equivalent BMIs of 26.8 kg/m^2^ and 27.3 kg/m^2^, respectively. We did not identify differential conversion rates secondary to obesity; however, there is insufficient data to draw firm conclusions on this topic.

Total operative time, warm ischemia time, and length of stay are surrogate measures of outcome. Warm ischemia time was the only operative parameter that was significantly different between the LDN and HALDN groups, with shorter WIT reported in HALDN procedures. Investigators have attributed this difference to the increased tactile control in HALDN, leading to faster vessel management and kidney extraction [3, 6, 7, 13, 40]. We did not find any statistical difference for either total operative time or length of hospital stay between the techniques. The studies that directly compared the latter two variables in LDN and HALDN procedures reported conflicting trends. It was difficult to compare total operative time with confidence because investigators do not always report a uniform end point that would allow a direct a comparison between institutions. Despite this limitation we observed a wide range in total operative times for each technique (LDN 78.4–253 minutes; HALDN 83–283 minutes). This suggests that center-specific practices and/or experiences influence the operative time reported in the literature. 

Our data summarizes the rates of complications and operative statistics reported in the peer-reviewed literature of large institutional studies. Therefore, there are limitations to our study. We have no resources to test the validity of the published findings or identify all the center specific variables that determined the reported outcome. We therefore cannot guarantee that the observations calculated from the summated data can be generalized to other transplant centers. Our conclusions are therefore limited to the specific dataset that was analyzed. While the datasets include a large number of patients, there could be a systematic bias associated with restricting our search to published studies. The use of publications with diverse study designs prevented us from using meta-analysis. Thus, we used simple observational outcomes from the published peer-reviewed literature to create a dataset for analysis and did not use a common measure of effect size. Because we were unable to control the effects of all study characteristics, the dataset incorporates several sets of assumptions and conditions. Even though the data set must be interpreted with caution, it provides a large compendium of outcome information as a first step in assessing performance for quality outcome purposes.

At present there is no evidence that proves one laparoscopic technique is superior to the other. There are however consistent trends in the data suggesting that intraoperative injuries are more common in LDN patients while post-operative injuries are more common in HALDN donors. Analysis of major donor morbidity differentiates the two techniques. Transfusions and conversion to open procedures from vascular injury are the most common sources of major donor morbidity reported in the literature. These are cited more frequently in LDN procedures. Conversely, there did not appear to be a difference in the overall rate of major post-operative complications. As such, this paper suggests that the HALDN approach has less associated risk of major donor morbidity.

## Figures and Tables

**Table 1:** Study characteristics, donor demographics and intraoperative data.

Author et al.								Total				Warm			
	Study	Cases		Age		BMI		operative		Operative		ischemia		Length of	
	type			(years)		(kg/m2)		time		blood loss		time		stay	
								(min)		(mL)		(min)		(days)	
		L	H	L	H	L	H	L	H	L	H	L	H	L	H

Anderson [14]	R		103		34.4		25.9		283				2.3		3.7
Bargman [15]	RCT	20	20					200	219	141.5	97.4	2.6	2.3	1.9	2.1
Branco [16]	R	89	67	38.9	38			78.4	83	98.9	130.9	2.5	3.6	1.4	2.8
Breda [17]	R	300		36.7		28.3		180		80		4.0			
Chandak [18]	P		144		44				198		160		2.9		
Chin [9]	R	500		40.3		27.3		208.2		197		3.5		2.3	
Desai [19]		303		45.2		24.2		159.0				6.5		4.4	
Diner [20]	R	167		39.0				226.3		108.3		5.0		2.5	
Dolce [21]	P		217		39.6				177.3		71.1		1.1		3.6
Dols [22]	P	283		50				222		206		5.6		3.5	
El-Galley [23]	P		80		43				184		50		4.0		
Fettouh [24]	R	400		32				117*		56		2.6			
Fisher [25]	R		200		42		27		229		243		2.6		1.9
Giron [26]	R		85		34.3				132		125		4.0		2.5
Gupta [27]	P	343		43				191				3.3		3.7	
Hawasli [28]	R	168						132		92		3.5		1.2	
Heimbach [29]	R		553		42		28.0		137*						2.3
Husted [30]	R	213	40	41	41	27.5		192	186			3.3	3.9	1.9	1.9
Jacobs [31]	R	738		40.2		27.7		202.1		128		2.8		2.7	
Jeon [32]	R	71	12	36	38			206.7*	143.4*			3.8	2.4		
Keller [33]	R		230						176		71		1.1		3.7
Ko [34]	R	400		40.9		27.1		147.6		83.2		1.5		2.0	
Kocak [35]	R	482	318	39	41	27	29							1.6	1.2
Lai [36]	P	12	12	48.6	44	22.5	25	215	258			4.5	3.8	5.8	5.6
Lallas [37]	R	230		40.6				112		92.8				2.3	
Li [38]	R		65						157.92		37.38		3.3		5.0
Melcher [39]	R	530		40.4		26.1		196						3.2	
Minnee [40]	P		158		46.7				173.8				3.2		4.9
Percegona [41]	R	34	21					184	191	441	545	3.8	4.3	2.6	3.6
Permpongkosol [42]	R	553		41.4											
Rajab [43]	R		80		38		26.5								
Ruszat [44]	R	12	33	46	50	24	24	212	192	307	208	4.0	2.1	13.0	11.0
Salazar [45]		11	24	39	44	26	26	213	235					3.0	4.0
Seo [46]	R		100		38.1		23		202				3.0		4.1
Simforoosh [47]	P	241		27.8		24.8		136.5				7.5			
Su [48]	R	381						253		334		4.9		3.3	
Sundaram [49]	R	253		39.3		26.1		199		115		2.2		2.8	

Total		6734	2562												
Weighted mean				39.9	41.2	26.8	27.3	187.0	189.3	144.0	128.6	3.7	2.5	2.7	3.0

Difference (L–H) (95% CI)				−1.3(−4.2, 1.7)		−0.5(−1.8, 0.8)		−2.3(−31.4, 26.7)		15.4(−65.1, 95.8)		1.2(0.1, 2.3)		−0.3(−1.1, 0.6)	
*P*-value				.401		.426		.876		.711		**.037** ^†^		.547	

R:Retrospective; P:Prospective; RCT:Randomized Control Trial; L:LDN; H:HALDN.

*Excluded from weighted mean calculation (alternative endpoints); ^†^ *P* < .05.

**Table 2:** Intraoperative complications of LDN and HALDN.

	LDN		HALDN		*P*-value
Bowel injury	27	0.4%	3	0.1%	**.031** ^†^
					
Liver/mesenteric/splenic injury	48	0.7%	4	0.2%	**.001** ^†^
					
Pulmonary injury					
Atelectasis	2	<0.0%	0	0.0%	.383
Diaphragm injury	4	0.1%	0	0.0%	.217
Pneumothorax	10	0.1%	0	0.0%	.051

	16	0.2%	0	0.0%	.014^†^
Renal/ureteral injury					
Bladder injury	5	0.1%	1	<0.0%	.550
Degloving of the kidney capsule	2	<0.0%	2	0.1%	.315
Renal laceration	9	0.1%	0	0.0%	.064
Ureteral injury	10	0.1%	2	0.1%	.398

	26	0.4%	5	0.2%	.154
Vascular injury/bleeding					
Bleeding-vascular injury	97	1.4%	24	0.9%	.056
Major bleeding (> 2 UPRBCs or > 500 mL)	35	0.5%	3	0.1%	**.007** ^†^

	132	2.0%	27	1.1%	**.003** ^†^
Procedural complications					
Conversion—Elective/obesity	17	0.3%	4	0.2%	.382
Conversion—reason not reported/other	19	0.3%	5	0.2%	.460
Difficult manual extractions/entrapment sack malfunction	12	0.2%	0	0.0%	**.033** ^†^
Endotracheal intubation/extubation difficulties	3	<0.0%	0	0.0%	.285
Staple misfire	4	0.1%	0	0.0%	.217

	55	0.8%	9	0.4%	**.015** ^†^
Other complications					
Cardiac arrhythmias	2	<0.0%	0	0.0%	.383
Hydrocele	2	<0.0%	0	0.0%	.383
Transient CO2 pneumoperitoneum	2	<0.0%	0	0.0%	.383
Other	40	0.6%	2	0.1%	**.001** ^†^

	46	0.7%	2	0.1%	**<.001** ^†^
					
Total intraoperative complications	350	5.2%	50	2.0%	**<.001** ^†^

^†^ *P* < .05					

**Table 3:** Postoperative complications of LDN and HALDN.

	LDN		HALDN		*P*-value
Abdominal complications					
Chylous ascites	5	0.1%	4	0.2%	.257
Other	10	0.1%	11	0.4%	**.011** ^†^
	15	0.2%	15	0.6%	**.006** ^†^
Bowel complications					
Ileus	35	0.5%	26	1.0%	**.008** ^†^
Intestinal obstruction	9	0.1%	2	0.1%	.486
Other	1	<0.1%	1	<0.1%	.478

	45	0.7%	29	1.1%	**.025** ^†^
Cardiac complications					
Hypertension requiring medication	11	0.2%	0	0.0%	**.041** ^†^
Myocardial infarction	1	<0.0%	0	0.0%	.537
Other	2	<0.1%	1	<0.1%	.823

	14	0.2%	1	<0.0%	.070
Incision/wound complications					
Incisional hernia	10	0.1%	18	0.7%	**<.001** ^†^
Incisional, back or abdominal pain	12	0.2%	9	0.4%	.116
Wound infection	91	1.4%	50	2.0%	**.034** ^†^
Other	3	<0.1%	3	0.1%	.218

	116	1.7%	80	3.1%	**<.001** ^†^
Neural complications					
Extremity neuropathy	13	0.2%	3	0.1%	.430
Paralysis, emesis, vomitus	4	0.1%	2	0.1%	.752

	17	0.3%	5	0.2%	.611
Pulmonary complications					
Pneumonia	13	0.2%	4	0.2%	.710
Other	7	0.1%	1	<0.1%	.340

	20	0.3%	5	0.2%	.397
Renal/ureteric complications					
Urinary retention	12	0.2%	19	0.7%	**<.001** ^†^
Urinary tract infection	12	0.2%	19	0.7%	**<.001** ^†^
Other	5	0.1%	2	0.1%	.952

	29	0.4%	40	1.6%	**<.001** ^†^
Vascular/hematoma complications					
Continued bleeding	13	0.2%	0	0.0%	**.026** ^†^
Hematoma	28	0.4%	8	0.3%	.473
Pulmonary embolism/DVT	0	0.0%	5	0.2%	**<.001** ^†^
Subcapsular hematoma	13	0.2%	0	0.0%	**.026** ^†^
Other	5	0.1%	6	0.2%	**.045** ^†^

	59	0.9%	19	0.7%	.525
					
Other complications	135	2.0%	26	1.0%	**.001** ^†^
					
Total postoperative complications	450	6.7%	220	8.6%	**.002** ^†^

^†^ *P* < .05					

**Table 4:** Major intraoperative and postoperative complications of LDN and HALDN.

	LDN		HALDN		*P*-value
Intraoperative complications					
Aborted procedures					
Colon injury	1	<0.1%	0	0.0%	.537
Mesenteric vein injury	1	<0.1%	0	0.0%	.537

	2	0.0%	0	0.0%	.383
					
Blood transfusions	51	0.8%	6	0.2%	**.004** ^†^
					
Conversions					
Bleeding	55	0.8%	11	0.4%	**.047** ^†^
Elective/obesity	15	0.2%	4	0.2%	0.525
Reason not reported/other	19	0.3%	5	0.2%	0.460

	89	1.3%	20	0.8%	**.030** ^†^
					
Other					
Bowel resection	1	<0.1%	0	0.0%	0.537
Splenectomy	4	0.1%	2	0.1%	0.752

	5	0.1%	2	0.1%	0.952
					
Total intraoperative complications	147	2.2%	28	1.1%	**.001** ^†^
					
Post-operative complications					
Re-hospitalizations					
Ileus	3	<0.1%	5	0.2%	**.027** ^†^
Infection	1	<0.1%	1	<0.1%	.478
Pain	0	0.0%	1	0.0%	.105

	4	0.1%	7	0.3%	**.007** ^†^
					
Re-operations					
Bleeding/hematoma	10	0.1%	1	<0.1%	.170
Bowel injury	2	<0.1%	1	<0.1%	.823
Incisional hernia	4	0.1%	0	0.0%	.217
Small bowel obstruction	6	0.1%	3	0.1%	.698
Other	4	0.1%	4	0.2%	.155

	26	0.4%	9	0.4%	.807
					
Death					
Myocardial infarction	1	<0.1%	0	0.0%	.537
Pulmonary embolism	0	0.0%	1	<0.1%	.105

	1	0.0%	1	0.0%	.478
					
Total post-operative complications	31	0.5%	17	0.7%	.222
					
					
Total major complications	178	2.6%	45	1.8%	**.013** ^†^

^†^ *P* < .05					
